# Characterization and evaluation of the efficacy of phage E21 therapy in a wound animal model of biofilm-associated *Pseudomonas aeruginosa* infection

**DOI:** 10.1038/s41598-026-52857-1

**Published:** 2026-05-19

**Authors:** Ahmed M. Salah Eldin, Ahmed S. Abu Zaid, Rania Ibrahim Shebl, Mahmoud A. Yassien

**Affiliations:** 1 Department of Microbiology and Immunology, Faculty of Pharmacy, Ahram Canadian University (ACU), 6th October, Giza, Egypt; 2 Department of Microbiology and Immunology, Faculty of Pharmacy, Ain Shams University, Organization of African Unity St. Abbassia, POB: 11566, Cairo, Egypt

**Keywords:** Skin infection, *Pseudomonas aeruginosa*, Strong biofilm, Phage therapy, Carboxy methylcellulose, Biotechnology, Microbiology

## Abstract

Skin infections caused by strong biofilm *Pseudomonas aeruginosa (P. aeruginosa)* are considered a serious public health issue because of the increased resistance toward the currently available antibiotics. Consequently, innovative therapeutic strategies have emerged to address these challenging infections. Among them, phage therapy stands out, in which highly potent lytic bacteriophages (phages) are specifically selected to target and eradicate the responsible pathogens. In this study, Pseudomonas phage E21 was recovered from sewage, and it genetically belongs to the *Lavrentievirus* genus, *Casjensviridae* family. The genetic characterization of the isolated phage reveals the presence of highly potent lytic enzymes, which play a critical role in effectively suppressing the growth of the targeted pathogens. The phage has high stability patterns over a wide range of temperatures and pH values (65 ℃ and 3–11). Carboxymethylcellulose was used to formulate a hydrogel for the evaluation of the bacteriophage’s efficacy against biofilm-associated wound infection in a suitable animal model. The result of the preclinical study confirmed the efficacy of isolated phage in the therapy of biofilm-associated wound infection.

**Supplementary Information:**

The online version contains supplementary material available at 10.1038/s41598-026-52857-1.

## Introduction

There is no doubt that the treatment of skin and soft tissue infections (SSTIs) caused by Gram-negative bacteria is more challenging due to the increasing rise in antibiotic resistance. *P. aeruginosa* is one of the most clinically significant Gram-negative bacteria. *P. aeruginosa* is ranked third among the top Gram-negative pathogens associated with healthcare-associated infections (HAIs), as reported by the National Healthcare Safety Network (NHSN).

*P. aeruginosa* causes a broad spectrum of infections, ranging from surgical site infections to deeper infections like pneumonia, bloodstream infections, and urinary tract infections. Also, the highly significant morbidity and mortality rates were associated with an elevated prevalence of antimicrobial resistance^1^. This recorded resistance is strongly related to *P. aeruginosa* biofilm formation potential. Biofilm is an extracellular polymeric matrix that is mainly formed of polysaccharides. This formed polysaccharide matrix aids the pathogen to establish the infection by allowing the bacterial cells to communicate with each other, decreasing the penetration of the antimicrobial agents, in addition to evading the human immune system. Consequently, the most critical issue for researchers and healthcare providers is to search for alternatives to the currently available antimicrobial agents to overcome the problem of antimicrobial resistance^2^.

Phages have been considered one of the most popular alternatives to antibiotics. They are capable of eliminating bacterial infections by infecting the bacterial host cell, leading to bacterial cell lysis. Typically, phages are distinguished by their ability to target specific bacterial species. This selectivity depends on the presence of specific receptors on the bacterial cell, to which the phage irreversibly binds. Additionally, the specific activity of the phage toward bacterial cells and inert activity toward human cells is explained by the fact that phages search for and bind only to receptors found exclusively on bacterial cells and absent on eukaryotic cells. Furthermore, phages are equipped with tails that perform crucial functions in infecting bacterial cells, including producing depolymerase enzymes with various roles. These enzymes target the bacterial cell, inducing lysis, and also target virulence factors such as biofilms and capsular polysaccharides. The combined action of these enzymes is to inhibit bacterial virulence factors and promote complete binding between the phage and the bacterial host cell, leading to lysis. The history of Pseudomonas phages and their use in wound infection models was declared to have high potential for conducting more studies, as McVay et al., who noticed the highest reduction of the *P. aeruginosa* bacterial cells in the wound site and decreased mortality after the treatment with phage cocktail^3^. Other newer studies emphasized the successful use of phage therapy on the skin infection, as Ma et al., who discovered a novel phage vB_PaeP_PZH3, against MDR *P. aeruginosa*, with a high therapeutic outcome in a mouse wound infection model^4^. Therefore, the current study focuses on the isolation and characterization of phages with promising antibacterial and antibiofilm potentials against a highly biofilm-producing *P. aeruginosa* clinical isolate recovered from infected wounds.

## Results

### Collection and identification of clinical isolates

Forty clinical isolates were identified as *P. aeruginosa* by the appearance of green-to-blue colonies upon culturing on cetrimide agar, in addition to positive catalase and urease reactions. All data regarding the characterization of the clinical isolates were summarized in the supplementary file (Table S1).

### Quantitative determination of bacterial biofilm formation

According to the recorded results, the ODc = 0.09; therefore, out of the forty collected isolates, 7.2% (*n* = 3) were classified into weak biofilm producers, whereas 17.5% (*n* = 7) were moderate, while 75% (*n* = 30) of the recovered isolates showed strong biofilm production. The isolates’ biofilm classification was added in the supplementary files (Table S1).

### Isolation of bacteriophages against the strong biofilm producer *P. aeruginosa*

#### Screening for the phage lytic activity and plaque assay

**Fig. 1:**
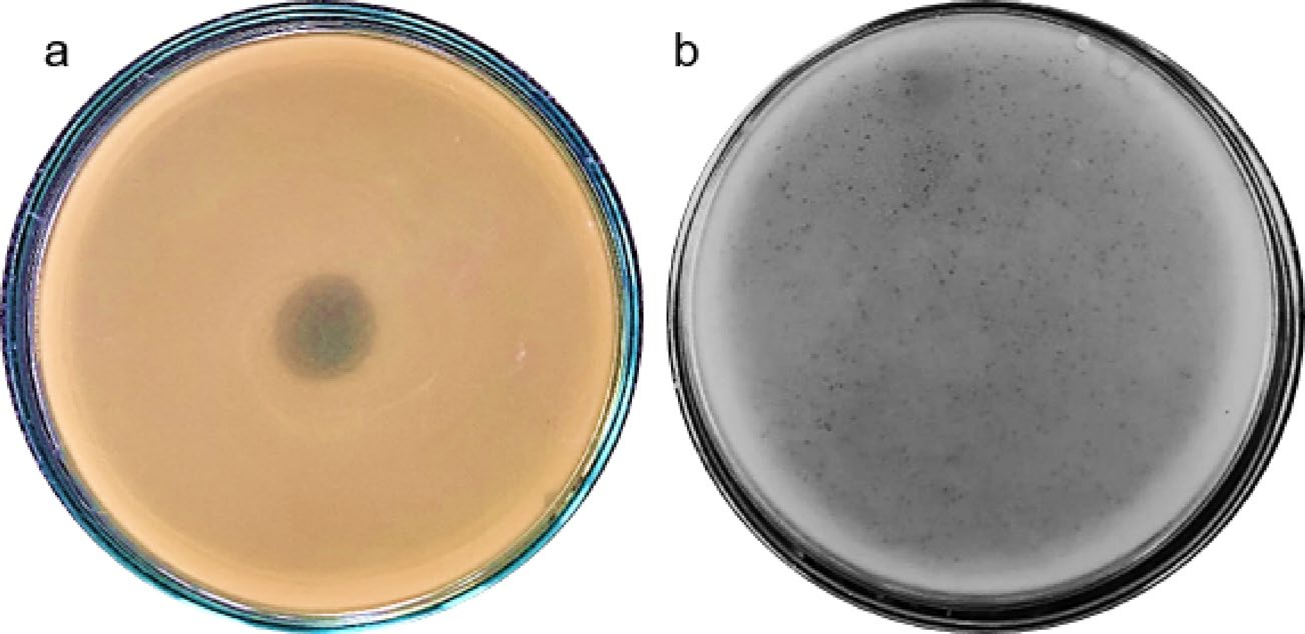
(**a**) Spot test of the isolated phage indicating lytic activity against *P*. *aeruginosa* 6PS isolate. (**b**) Plaque assay for the phage lysate demonstrating tiny circular clear plaques less than 1 mm.

Spot tests revealed that one out of seven samples showed lytic activity against the strong biofilm producer *P. aeruginosa* 6PS (Fig. 1a). Plaque assay demonstrated that the initial titer of the phage lysate was 1.2 × 10^8^ PFU/ml. The morphological appearance of the plaques demonstrated small, clear plaques (about 1 mm in diameter) with circular, regular edges (Fig. 1b). (The uncropped pictures of Fig. 1a and b were added in the supplementary file as Fig. S1 and Fig. S2)

### Characterization of the isolated phages showing lytic activities against *P. aeruginosa*

#### Morphological characterization

**Fig. 2:**
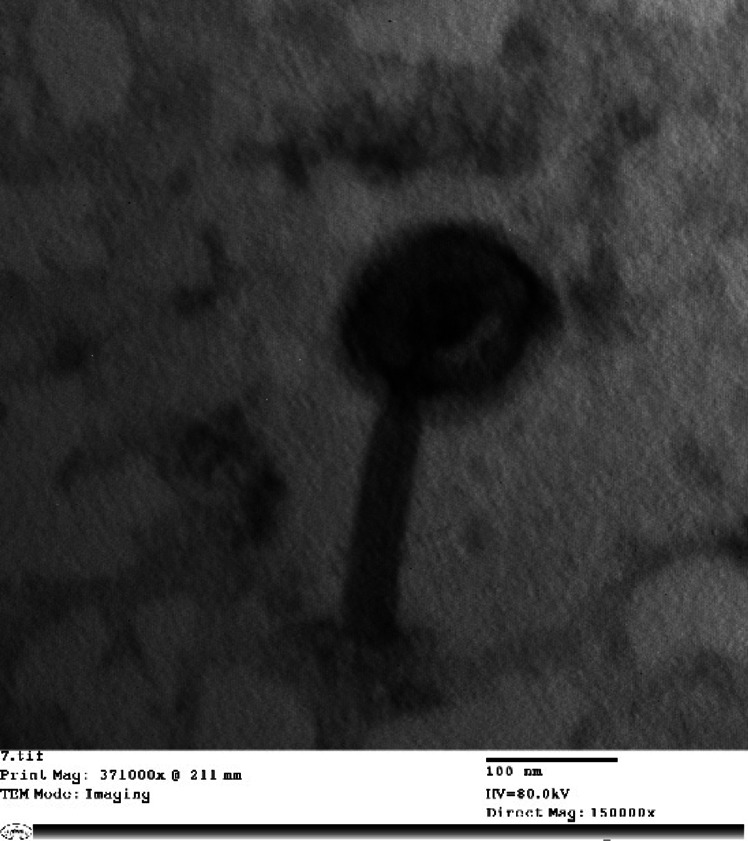
Transmission electron microscopic images of the isolated phage. Scale bar represents 100 nm.

Imaging by TEM suggested that the phage lysate sample is composed of one phage (Pseudomonas-E21). Phage Pseudomonas-E21 showed an icosahedral head (84.3 nm approximately) with a contractible tail of 94.5 nm length and 20.2 nm width (Fig. 2). These observations matched those of the “Viral Zone” website, along with the guidelines provided by the International Committee on Taxonomy of Viruses (ICTV), which indicated that Pseudomonas phage -E21 belongs to the *Casjensviridae* family.

#### Complete genome sequencing of *Pseudomonas phage* E21

Results of the complete genome sequencing of the phage lysate demonstrated the presence of Pseudomonas phage-E21, which carries a genomic DNA size of 58,554 bp, and taxonomical classifications based on BLASTn alignment analysis showed that the phage belongs to the *Lavrentievavirus* genus, *Casjensviridae* family. The whole genome sequence of the isolated phages was submitted to the NCBI GenBank (National Center for Biotechnology Information), and their accession number was as follows: Pseudomonas phage E21 (PV596763). The whole genome sequence was annotated and expressed as a circular genome map. The number and positions of the ORFs of Pseudomonas phage -E21 are listed in supplementary files in Table S2.

**Fig. 3:**
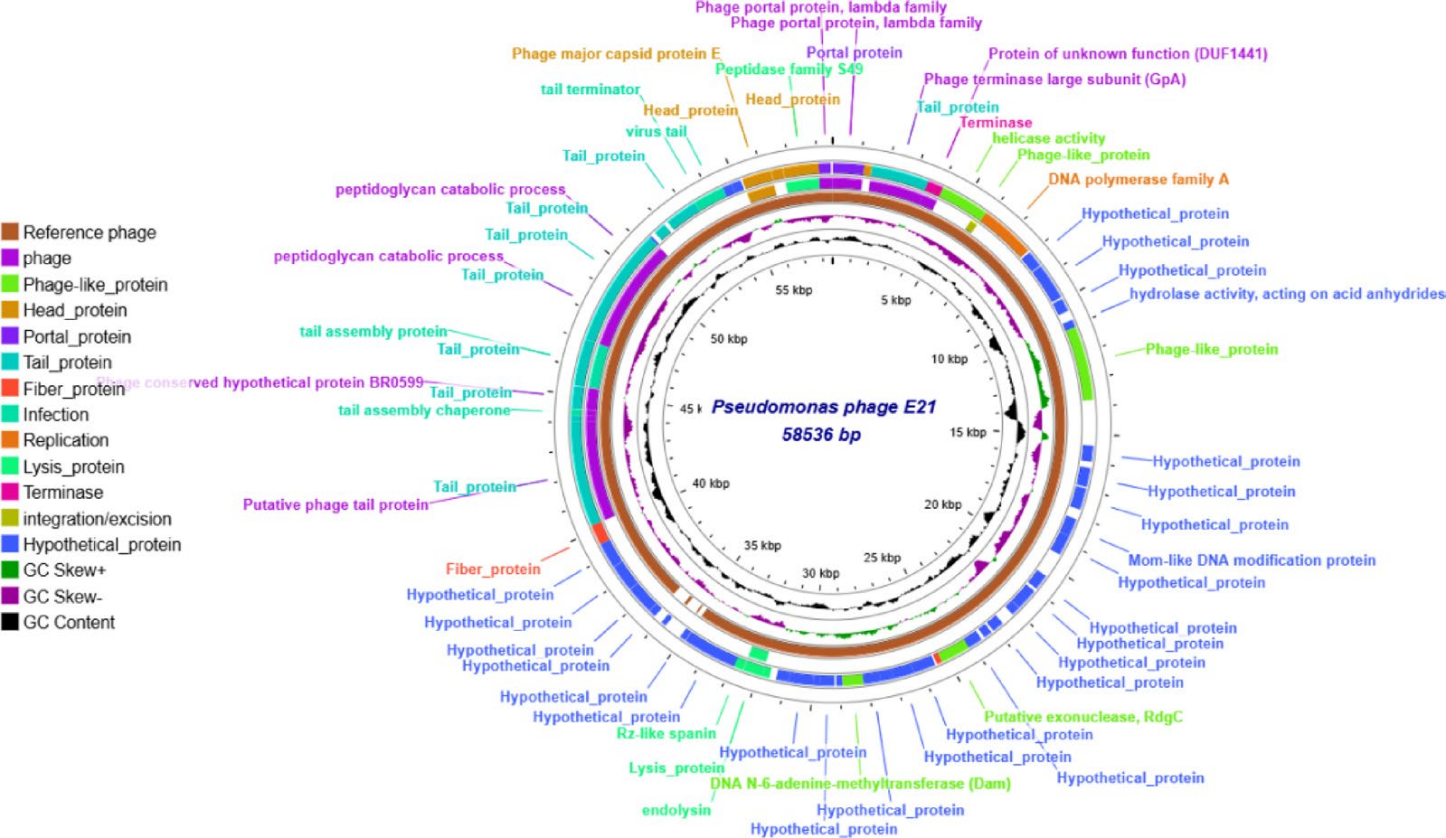
Circular genomic maps of Pseudomonas phage E21, and the annotated ORFs of the phages, displaying their genomic allocation along the whole genome sequence. Generated by proksee Proksee - Genome Analysis.

The circular genome maps and the annotated ORFs of Pseudomonas phage -E21 are depicted in Fig. 3.

#### Host range

The isolated phage demonstrated potential lytic activity against 7 isolates out of the total isolated strong biofilm producers, *P. aeruginosa* isolates (Table 1).

**Table 1:** Host range of Pseudomonas phage E21 against the strong biofilm producers’ *P. aeruginosa* isolates, along with biofilm production and antibiotic susceptibility pattern.

Isolate code	Spot test	Biofilm		Susceptibility pattern						
		OD	Classification	AT	CPM	MRP	PI	PIT	GEN	LEV
1PS	**-**	0.837 ± 0.026	Strong	R	R	S	R	R	R	R
2PS	**-**	0.888 ± 0.043	Strong	R	R	R	R	R	R	R
3PS	**-**	0.950 ± 0.040	Strong	R	R	R	R	R	R	R
4PS	**-**	0.898 ± 0.034	Strong	R	R	R	R	R	R	R
6PS	**+**	0.991 ± 0.024	Strong	R	S	R	R	R	R	R
7PS	**-**	0.968 ± 0.021	Strong	R	R	R	R	R	R	R
8PS	**-**	0.875 ± 0.040	Strong	R	R	R	R	R	R	R
11PS	**+**	0.889 ± 0.018	Strong	R	R	R	R	S	R	R
12PS	**-**	0.953 ± 0.047	Strong	R	R	R	R	R	R	R
15PS	**-**	0.938 ± 0.024	Strong	R	R	R	R	R	R	R
16PS	**-**	0.859 ± 0.022	Strong	R	R	R	R	R	R	R
18PS	**-**	0.790 ± 0.028	Strong	R	R	R	R	R	R	R
20PS	**+**	1.090 ± 0.031	Strong	R	R	R	R	R	R	R
21PS	**-**	0.360 ± 0.014	Strong	R	R	R	R	R	R	R
22PS	**-**	0.400 ± 0.010	Strong	R	R	R	R	R	R	R
23PS	**+**	0.941 ± 0.027	Strong	R	R	R	R	R	R	R
24PS	**-**	0.916 ± 0.028	Strong	R	R	R	R	R	R	R
25PS	**-**	0.700 ± 0.024	Strong	R	R	R	R	R	R	R
26PS	**-**	0.841 ± 0.037	Strong	R	R	R	R	R	R	R
27PS	**-**	0.991 ± 0.026	Strong	R	R	R	R	R	R	R
28PS	**+**	0.873 ± 0.031	Strong	R	R	R	R	R	R	R
29PS	**-**	0.920 ± 0.035	Strong	R	R	R	R	R	R	R
31PS	**+**	0.500 ± 0.019	Strong	R	R	R	R	R	R	R
33PS	**-**	0.845 ± 0.019	Strong	R	R	R	R	R	R	R
34PS	**-**	0.934 ± 0.045	Strong	R	R	R	R	R	R	R
35PS	**-**	0.957 ± 0.047	Strong	R	R	R	R	R	R	R
36PS	**-**	0.891 ± 0.039	Strong	R	R	R	R	R	R	R
37PS	**+**	0.960 ± 0.028	Strong	R	R	R	R	R	R	R
38PS	**+**	0.985 ± 0.023	Strong	R	R	R	R	R	R	R
39PS	**-**	0.905 ± 0.037	Strong	R	R	R	R	R	R	R

Host range activity of Pseudomonas phage E21 against *P. aeruginosa* isolates. Lytic activity was determined by spot assay and recorded as (+) indicating lysis and (–) indicating no lysis. Biofilm production is presented as mean ± SD. Antibiotic susceptibility profiles are expressed as S (susceptible) and R (resistant) based on CLSI criteria. The antibiotics tested were aztreonam (AT), cefepime (CPM), meropenem (MRP), piperacillin (PI), piperacillin/tazobactam (PIT), gentamicin (GEN), and levofloxacin (LEV).

#### Antibiofilm potential of the isolated phage

**Fig. 4:**
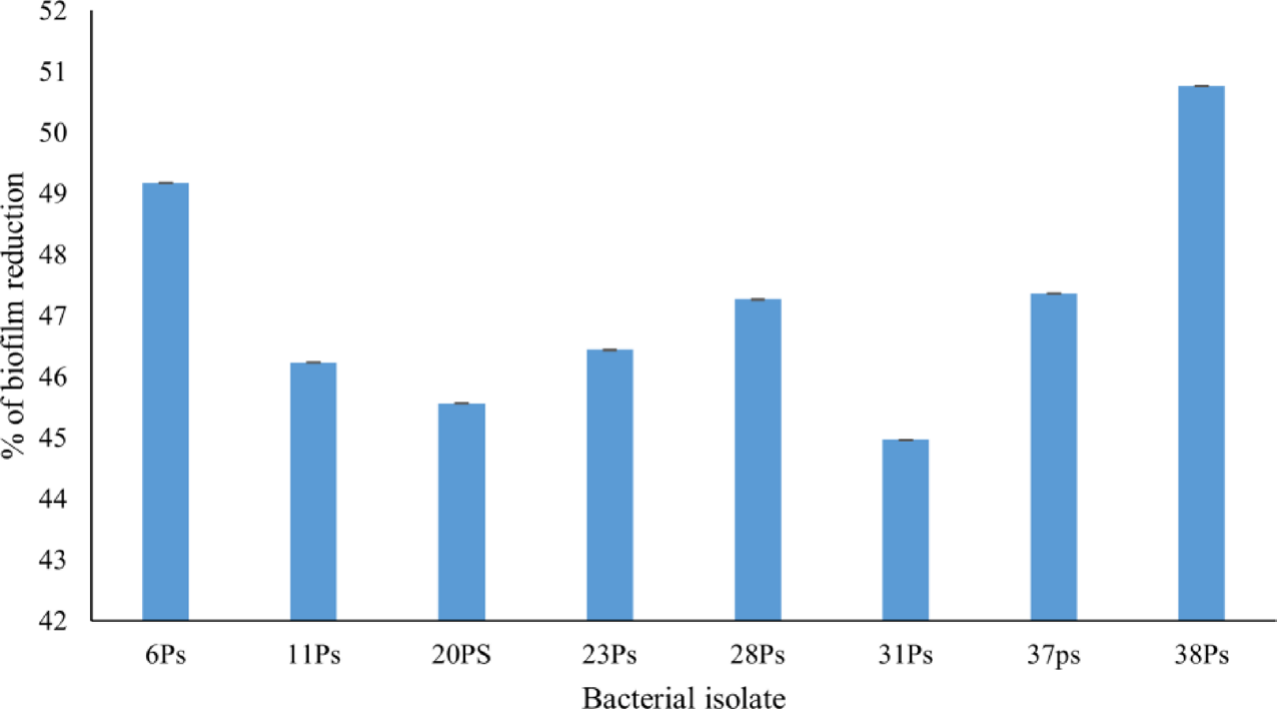
Antibiofilm potentials of Pseudomonas phage E21 against eight strong biofilm-forming *P. aeruginosa* isolates. Data were presented as means ± standard deviations (SD).

The anti-biofilm activity of Pseudomonas phage E21 lysate against eight strong biofilm producer isolates was expressed as a percentage reduction in biofilm formation. The results showed that treatment with the isolated phage caused a partial reduction inbiofilm formation, ranging from 45% to 50%, as compared to the control (Fig. 4).

#### In vitro activity of the tested hydrogels

**Fig. 5:**
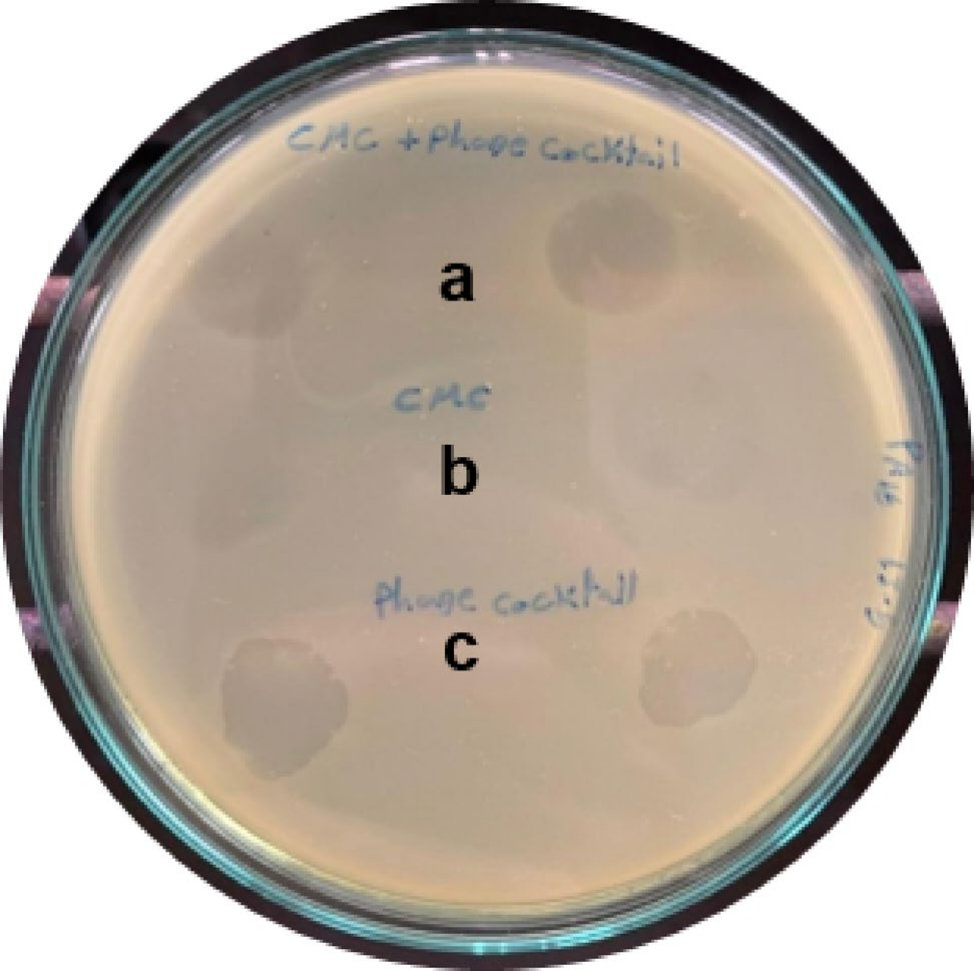
In vitro activity of the Pseudomonas phage E21-loaded hydrogel (**a**), negative control (**b**), and positive control (**c**) against a strong biofilm-forming *P. aeruginosa* clinical isolate.

An obvious inhibition zone was observed in the case of Pseudomonas phage E21-loaded hydrogel (a) as well as in the case of the positive control of the phage lysate (c). On the other hand, the plain hydrogel negative control (b) didn’t show any activity (Fig. 5). The hydrogel maintained the lytic activity of the Pseudomonas phage E21for three months. (The uncropped picture of Fig. 2 was added in the supplementary file as Fig. S3).

#### In vivo potential of the phage hydrogel

Recorded data revealed that the survival rate for all rats at the end of the experiment was 100%.

#### Wound area closure

**Fig. 6:**
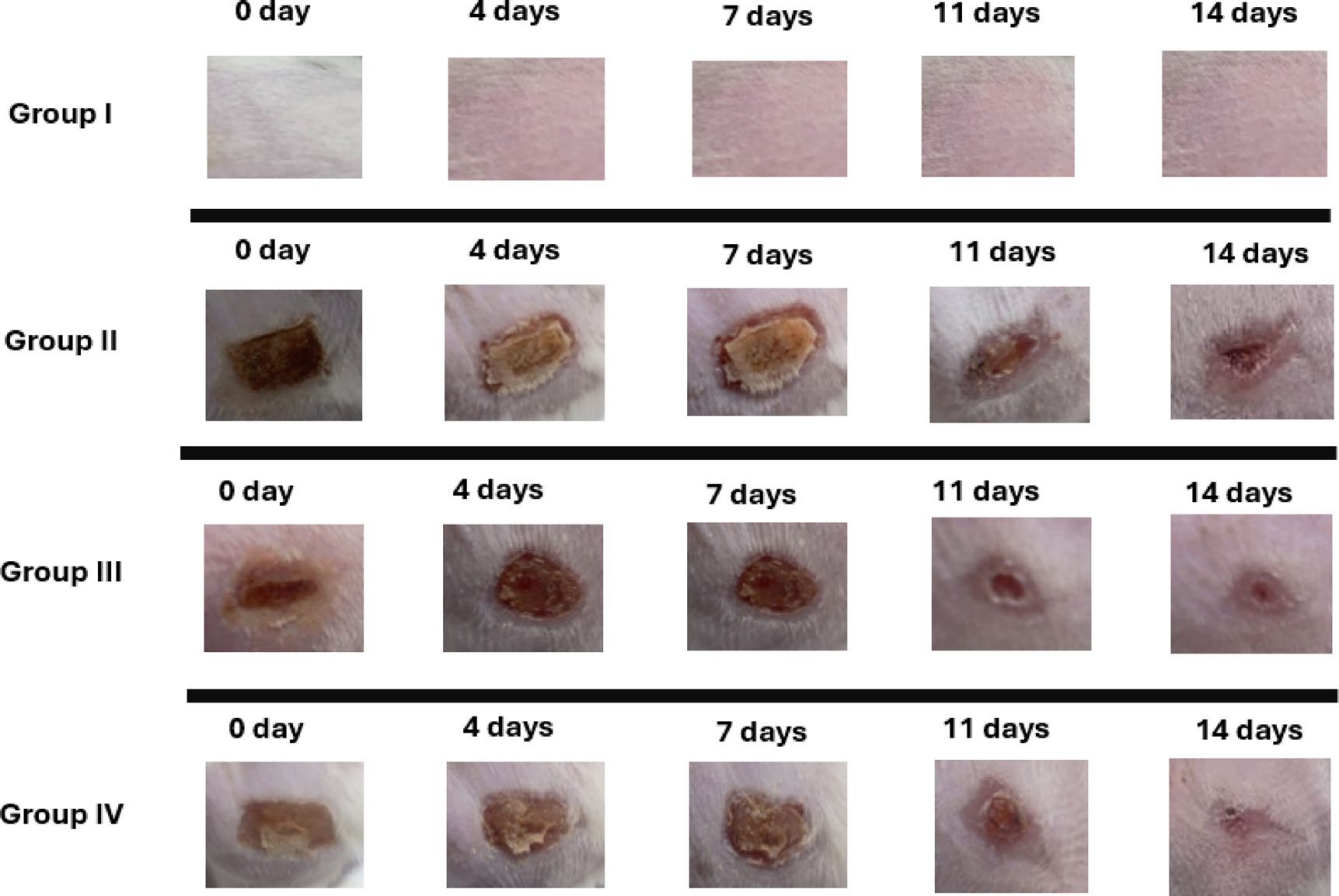
Photodocumentary assessment of the degree of wound healing in the *P. aeruginosa* infected groups at different time intervals (days 0, 4, 7, 11, 14).

Photographic documentation over 14 days demonstrated clear differences among the experimental groups (Fig. 6). Infection state, pus accumulation, inflammatory response, and the progress of the wound healing were the factors that were assessed along the in vivo study. On day 2, the development of infection at the wound site was observed visually in all the infected groups, while the normal (uninfected) group showed no abnormalities. On day 7: The normal group remained unburned, unaffected. In Group II (infected, untreated), the wound abnormalities remained unchanged with persistent signs of infection, which means no sign of tissue regeneration was expressed as 0% wound contraction. Group III (infected, treated with silver sulfadiazine) demonstrated noticeable pus accumulation, proposing a progressive inflammatory response, 30% wound contraction. In contrast, group IV (infected, treated with Pseudomonas phage -loaded hydrogel) showed noticeable progress in the healing process as indicated by 50% wound contraction. Finally, after 14 days, Group II continued to show pus accumulation without visible signs of healing, 40% wound contraction. Group III demonstrated diminished pus accumulation and more healing progression, 80% wound contraction. However, Group IV (burned, infected, treated with phage-loaded hydrogel) demonstrated further wound closure, which reached near total closure, 90%. These results show the significant differences between Group IV and Group III (burned, infected, treated with silver burn), p = **0.0036.** Evaluation of wound contraction was added in the supplementary files (Table S3).

Group I (normal group): non-infected, untreated; Group II: negative control, burned, infected, untreated; Group III: positive control, burned, infected, treated with silver burn cream. Group IV: Test, burned, infected, treated with phage-loaded hydrogel.

#### Bacterial clearance

The total bacterial viable count at the infected wound area was quantified using the viable count test, and the recorded data revealed a statistically significant reduction (*p* = 0.0026) in the bacterial count in group IV (infected, treated with phage -loaded hydrogel) as compared to group III (infected, treated with silver sulfadiazine) on day 14 supplementary files (Table S4).

#### Histopathology analysis

**Fig. 7:**
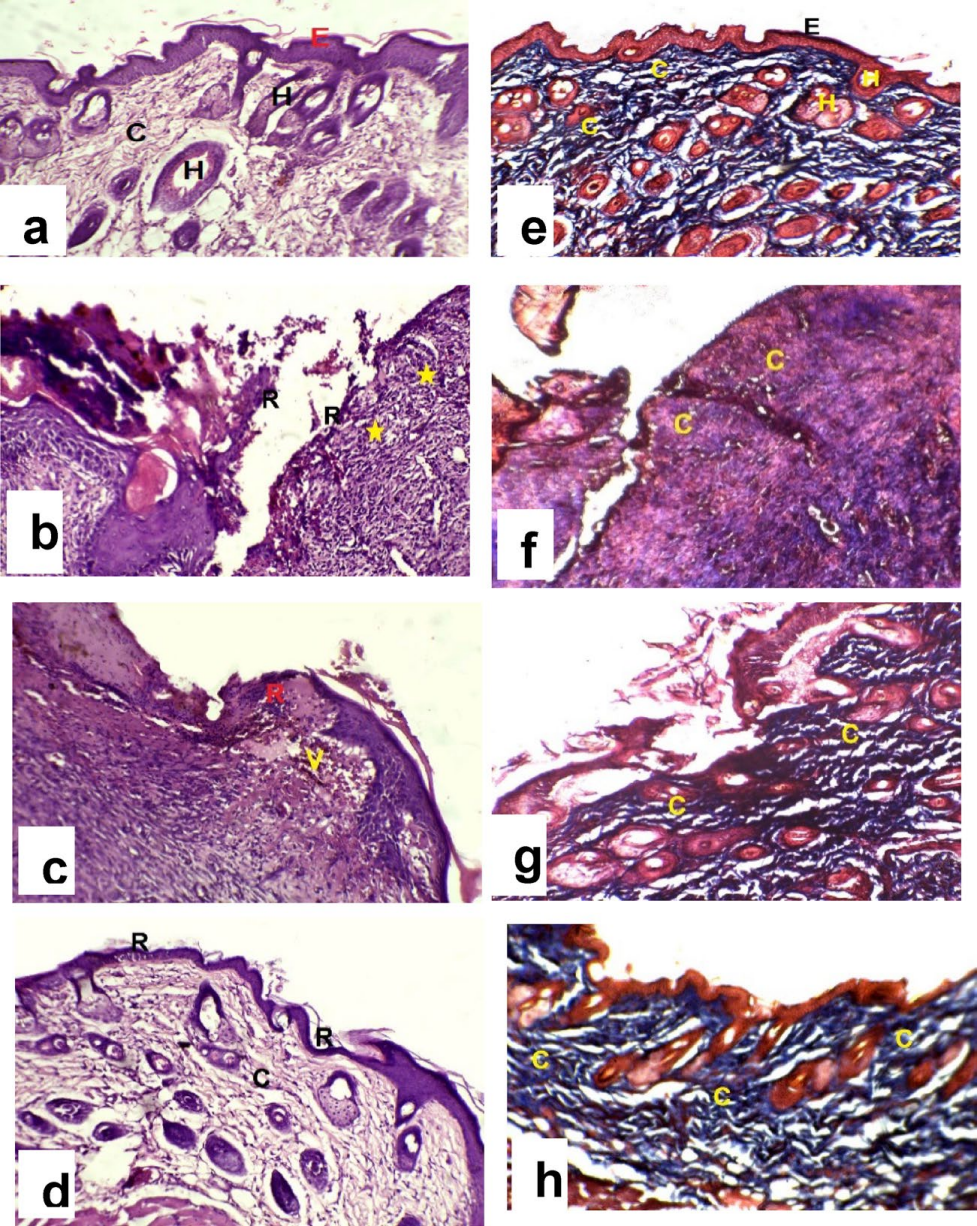
Histopathological analysis of four groups using H&E and Masson Trichrome staining techniques.

H&E imaging revealed that the granulation tissue in the *P. aeruginosa* -infected group was weakly organized after 14 days, exhibited an abundance of inflammatory cells, no re-epithelialization, and relatively few collagen fiber depositions (Fig. 7b) as compared to the uninfected group (Fig. 7a). The silver sulfadiazine-treated group exhibited moderate collagen deposition and reduced cellular infiltration, as well as regeneration of thin epidermis (Fig. 7c). Also, an incomplete epidermal layer was observed in rats treated with the Pseudomonas phage-loaded hydrogel after 14 days. Furthermore, the phage-treated granulation tissue exhibited elevated levels of fibroblast proliferation and dense, irregular collagen fibers in the dermis, as well as newly generated capillaries (Fig. 7d).

Masson Trichrome staining of Group I (Normal) (Fig. 7e) displays a healthy distribution of collagen fibers, accounting for 15.596% of the mean dermal area. In contrast, Group II (burned, infected, untreated) (Fig. 7f) displays a pathological surge in collagen deposition, reaching 43.623%; these fibers appear disorganized and excessively dense, characteristic of fibrotic scarring. Group III (burned, infected, treated with silver burn) (Fig. 7g) showed moderate improvement with a reduction in collagen area to 26.281%, though fibers remained somewhat irregular. Notably, Group IV (burned, infected, treated with phage-loaded hydrogel) (Fig. 7h) demonstrated the best recovery, with collagen levels at 19.698%. This value is nearer to the normal baseline, suggesting that the phage-loaded hydrogel promotes a more physiological healing process. The percentage of the area of collagen indicates that Group I (treated with phage–loaded hydrogel) has significantly higher efficacy than Group III, *P* = 0.000078. The Mean area percentage of collagen fibers of each group was added in the supplementary files, Fig. S4.

## Discussion

The highly progressive spread of hospital-acquired infection, especially that caused by the multidrug-resistant (MDR) *P. aeruginosa*, usually results in more challenging treatment^5^. In the meantime, the potential of *P. aeruginosa* to form biofilm, as one of its virulence factors, exacerbates the problem of antimicrobial resistance^6^. That is because the developed biofilm aids in building up a network between bacterial cells, which decreases the diffusion of the antimicrobial agents to the bacterial population. Moreover, this biofilm matrix facilitates the escape of the bacterial cells from the human immune cells^7^. Based on previous studies, the eradication of the bacterial biofilm could be achieved by enzymes that could cleave the biofilm matrix, resembling the enzymes released from the bacteriophages to target the bacterial biofilm^8^. Consequently, phage therapy is considered a reliable biological tool that could be developed to be an effective alternative to antibiotics^9^.

The phages selected for phage therapy must have a broad host range, in addition to being equipped with different groups of enzymes targeting important bacterial virulent factors, and facilitate the entry of the phage into the bacterial cells^10^. Phage therapy has proven to be effective in multiple clinical cases, particularly for patients with severe infections caused by MDR pathogens. For example, patients with systemic infections caused by MDR *Acinetobacter* sp., as well as pan-resistant *P. aeruginosa*, recovered following phage administration. However, more studies are needed in the in vivo studies for a further understanding of the appropriate use of phages as an effective therapy^11^.

The use of the limited host range phage was targeted toward a certain bacterial strain, which possesses a mechanical advantage because it offers precise antimicrobial action and lowers the activity of the phage toward normal flora. Also, it encourages the incorporation of the phage in a phage cocktail for broader activity^12^.

The present study targeted the investigation of the lytic and the anti-biofilm potentials of Pseudomonas phage formulated as carboxy methylcellulose (CMC)-loaded hydrogel in the treatment of the strong biofilm producer *P. aeruginosa*. The phage was isolated from one of the collected wastewater samples using one of the strong biofilm-producing *P. aeruginosa* isolates (6PS) as host cells. Spot test was used as a primary method to confirm the lytic activity, followed by plaque assay, which is a quantitative method for determining the phage titer, followed by purification of the phage lysate to reach a specific bacteriophage. Plaque assay revealed that the isolated phage has a relatively high and reproducible titer of > 10^8^ PFU/ml. The observed plaques were clear, circular with regular edges, small in size, about 1 mm, and encircled by halos. The host range results revealed that the isolated phage lysate was effective against eight strong biofilm-producing *P. aeruginosa* clinical isolates. Furthermore, evaluating the lytic activity of the phage lysate against different bacterial species is essential in future studies, knowing that phages that have limited activity against the beneficial normal flora would be preferable.

Classification of the isolated phage was carried out depending on its morphology by using TEM. The obtained result revealed the presence of one phage that belongs to the *Lavrentievavirus* genus, *Casjensviridae* family, which shares the same icosahedral capsids with double-stranded DNA and a phage tail.

Confirmation of the identification of the isolated phages was carried out by whole-genome sequencing. The result showed the presence of Pseudomonas phage-E21 carrying a genomic DNA size of 58,554 bp. The taxonomical classifications based on BLASTn alignment analysis showed that the isolated phage belongs to the Lavrentievavirus genus. The whole genome analysis of Pseudomonas phage-E21 allocates codons for a group of enzymes like Rz-like spanin and endolysin, which are predicted to show cleavage properties on the peptidoglycan of bacterial cells. Also, the genetic material of the isolated phage shares the presence of hydrolase and peptidase genes, which could be responsible for the lytic effect, which is essential for the complete clearance of the bacterial infection role in biofilm.

These enzymes work in harmony to successfully eradicate the bacterial infection and play a crucial role in biofilm clearance. The first one is the endolysin, which works on the degradation of the peptidoglycan layer and was found to suppress transcription of genes responsible for primary adhesion and exopolysaccharide production^13,14^. Rz-like spanin, which plays a role in cell lysis by the distribution of the outer membrane of the Gram’s negative bacterial cells through the fusion of the cell membrane with the outer membrane^8,15^. While the role of the hydrolase and peptidase is related to the natural occurrence of the biofilm structure, as the hydrolase enzymes work on the cleavage of the glycosidic bonds between the polysaccharide molecules present in the biofilm^16^. Also, the peptidase acts directly on the decomposition of the protein, which is a main component of the biofilm matrix^16^.

The importance of phage lytic activity, the host range, and stability studies were also essential for evaluating the effectiveness of the isolated phage. The isolated phage was effective against eight strong biofilm producers, *P. aeruginosa* isolates, which is a relatively acceptable moderate range in comparison with previous studies^17^. In which two lytic phages within the *Casjensviridae* family were isolated and characterized. The host range of both phages reveals the lytic activity against only one *P. syringae* isolate, which was not the original isolation host. Also, other studies have successfully isolated a phage with a wide host range, as^18^, in which Phage vB_Ps_ZCPS13 was isolated, and its host range demonstrates lysis of 28 out of the 30 tested *P. aeruginosa* isolates, which is a very wide host range.

The isolated phage should be formulated in a suitable formula in order to examine its in vivo activity. Carbopol polymer formula was evaluated as a phage carrier, but unfortunately, it showed poor ability to deliver the isolated phage upon in-vitro examination. These findings were different regarding the use of CMC polymer, which showed promising maintenance of the phage lytic activity^19^. It is characterized by its ability to form biopolymers with favorable properties, where it forms superabsorbent hydrogels with excellent water solubility and viscosity. CMC also provides a controlled phage release and adequate contact time following the hydrogel application, in addition to the availability of cellulose. This formulated hydrogel also maintains the phage lytic activity for three months at 4 °C. These findings were matched with previous studies that confirmed the stability of phage lytic activity when using CMC polymer^12^.

A rat thermal injury model was used for the pre-clinical evaluation of the formulated phage-loaded CMC hydrogel. After establishing infection with a strong biofilm-producing clinical isolate of *P. aeruginosa*, the hydrogel was applied directly to the burn site. The evaluated parameters were collagen content and healing of the wound. In order to get a reliable outcome, our findings were compared among the experimental groups. Group IV (infected and treated with phage-loaded-hydrogel showed visually significant lesion closure in comparison to group II (untreated), and group III (infected and treated with silver sulfadiazine). Also, the histopathological analysis showed that the wound infection by *P. aeruginosa* was vigorous and showed complete remodeling of the tissue organization. Regarding the effect of the treatment by silver sulfadiazine and phage-loaded hydrogel, there are complete differences among these groups. Group IV, which received phage-loaded hydrogel, demonstrated promising outcomes as the epithelialization of the tissues was markedly noticed in the level of fibroblast proliferation and dense collagen fibers deposition in the dermis layer, and an abundant neovascularization. Also, group III, which received silver sulfadiazine (reference drug available in the market), exhibited a moderation of the epithelization process that was noticed by the moderate collagen deposition, in addition to reduced inflammatory infiltration; however, tissue regeneration was initiated after 14 days of treatment. Group II, which was infected and untreated, demonstrated pus accumulation, no reduction in the inflammatory cells, and absence of tissue regeneration. Therefore, the treatment with the phage -loaded hydrogel was found to be promising in the treatment of such a type of resistant infection. Our findings were also supported by the results of the bacterial load clearance in group IV at the site of infection. These results prove the activity of the isolated phage against the examined bacterial clinical isolate, as indicated by a significant reduction in the bacterial viable count of group IV as compared to that in groups II and IV. Furthermore, the current study showed comparable results to other studies, where they reported that the isolated phage was found to be more effective compared to the standard antibiotic used in that study^12^. Also, our results are in agreement with other studies, even if the hydrogel polymer was different^20,21^.

Despite the promising findings, several limitations of this study should be highlighted. First, the moderate host range of our phage limited its broad applicability as an empirical therapy, where the broader host range suggested superiority over the moderate host range^22,23^. Second, our study evaluated a single-phage formulation; however, a phage cocktail may yield beneficial outcomes^23^., such as enhanced antibacterial efficacy and reduced development of new antibiotic resistance. Third, although the isolated phage showed moderate antibiofilm activity, its efficacy could be improved by optimizing its potency. Fourth, we didn’t examine the probability of the development of resistance against our phage; however, this point could be addressed in future studies. In the meantime, our study focused on the use of qualitative assessment for the initial characterization of the phage; however, the application of a quantitative assessment method, such as the efficacy of plating (EOP), provides a more promising approach for evaluating the lytic potential of the isolated phage.

In addition, the study didn’t assess pharmacokinetic aspects of the phage within the wound environment, including the phage persistence and distribution. Finally, no comparison was performed between the phage therapy and antibiotic–phage combination treatments, which have been shown to exhibit synergistic effects in some contexts. It is important to point out that these limitations are essential considerations for future research to further validate and optimize the therapeutic potential of this phage hydrogel.

## Conclusions

Pseudomonas phages -E21 belongs to the *Lavrentievavirus* genus, *Casjensviridae* family, and was isolated from a wastewater sample, purified, identified, and characterized. The isolated phage showed potential lytic activity against eight strong biofilm-forming *P. aeruginosa* isolates in vitro. The phage was incorporated into a CMC-hydrogel formulation for topical application to assess its in vivo therapeutic potential against strong biofilm producer isolates. The phage-loaded hydrogel resulted in a marked reduction in mortality among treated rats and significantly enhanced healing at the wound site, as evidenced by the histopathological examination and the recorded reduction in the bacterial viable count at the injury site. Therefore, the isolated phage could be suggested as a promising treatment option for these types of resistant infections; however, further clinical studies should be carried out to ensure the suitability of the respective phage hydrogels for clinical application in humans.

## Methods

### Collection and isolation of clinical isolates

Forty clinical specimens were collected from patients’ skin infections, such as open wounds, burns, abscesses, and pus. The isolated clinical isolates were inoculated into Tryptic Soy Broth (TSB) as an enrichment medium. The isolates were identified by streaking onto cetrimide agar and incubating overnight at 37 °C. Post incubation, the colonial morphological appearance and its characteristics were observed and checked. The clinical isolates were further identified by Gram staining and biochemical tests, including the oxidase and urease tests^24^. The clinical isolates were collected from Demerdash Hospital during routine microbiological sample collection in the hospital after the study was approved by the Faculty of Pharmacy, Ain Shams University, Ethics Committee, ACUC-FP-ASU REC# 339.

### Determination of the susceptibility profiles of the collected isolates

Following the recommendations stated by the Clinical Laboratory Standards Institute (CLSI) (8). The Kirby-Bauer disk diffusion method was applied for testing the antimicrobial susceptibility of the clinical isolates^25^. Briefly, bacterial suspensions with a turbidity equivalent to 0.5 McFarland standard adjusted spectrophotometrically were prepared from a freshly cultured clinical isolate. Each clinical isolate was streaked onto (4 mm) Mueller-Hinton agar plates using a sterile cotton swab in three different directions to ensure uniform growth. Antibiotic selection was based on CLSI guidelines. Aztreonam (AT), cefepime (CPM), meropenem (MRP), piperacillin (PI), piperacillin/tazobactam (PIT), gentamicin (GEN), and levofloxacin (LEV). Antibiotic discs were carefully placed onto the agar plates using sterile forceps. The plates were incubated overnight, and the susceptibility profiles were determined for the tested isolates according to the CLSI M100 guidelines (33rd edition, 2023).

### Quantitative determination of bacterial biofilm formation

Testing the potential for biofilm formation was applied to classify the obtained clinical isolates. Briefly, as described by Stepanović et al.^26^. Overnight culture was used for the preparation of bacterial suspension using tryptic soya broth supplemented by 1% glucose (TSBG); the optical density of the bacterial suspension was adjusted to be equivalent to 0.5 McFarland standard. Isolates were diluted 1:100 using TSBG, followed by dispensing 100 µL of the bacterial suspension into 96-well plates. Each isolate was tested in triplicate and incubated for 24 h at 37 °C to allow biofilm formation. Post incubation, the supernatant was gently discarded, followed by gentle washing with sterile phosphate buffer saline (PBS) in order to remove the planktonic cells. Biofilm fixation was carried out by subjecting the plate to 60 °C for 1 h. The formed biofilm was stained with 0.1% v/v crystal violet (CV) and incubated for 15 min at 37 °C, then the excess stain was removed by washing the plate twice with PBS. The attached stained biofilm was solubilized by applying 33% v/v acetic acid and incubated at 37 °C for 15 min. Finally, the optical density (OD) was measured at 630 nm using a microtiter plate reader (ELx800, Biotek, Winooski, VT, United States). Uninoculated TSB served as the negative control. The results were interpreted by calculating the OD cut-off values (ODc), and the isolates were reported as weak biofilm producers (ODc < OD ≤ 2 * ODc), moderate biofilm producers (2 * ODc < OD ≤ 4 * ODc), and strong biofilm producers (OD > 4 * ODc). The isolates that showed strong biofilm production were selected for phage isolation^27^.

### Isolation of bacteriophage against the strong biofilm producer *P. aeruginosa*

Seven wastewater samples were collected from the drainage systems of different hospitals. Samples were filtered using filter paper to remove coarse-sized suspended particles. Each sample was mixed with a freshly prepared strong biofilm bacterial isolate and a double-strength TSB in a ratio of 1:1:10, respectively, and supplemented with 10 mM CaCl_2_ and 1 M MgSO_4_. The mixture was incubated for 24 h at 37 °C. Following the incubation, the mixture was centrifuged for 20 min at 6000 rpm and filtered using a syringe filter (0.22 μm). The obtained supernatant was used as a phage lysate for the testing of the presence of phage with lytic activity^12^. The criteria for selection of the bacterial host isolates were based on the results of the calculated ODc values, and the isolates classified as strong biofilm producers were applied in phage isolation.

### Screening for the phage lytic activity and plaque assay

A spot test was carried out as a qualitative test for screening the phage lytic activity against the strong biofilm producers, *P. aeruginosa*, and then the phage titer was quantified using standard double agar overlay (DAO). Phage lysate was diluted 10-fold in saline-magnesium (SM) buffer. An equal volume of the phage lysate and the host suspension was mixed and allowed to stand for 10 min, followed by introducing the mixture to 3 mL soft overlay 0.7% TSA, and then poured over a previously prepared TSA layer. Post complete solidification, the plates were incubated at 28 °C overnight in an upright position. The phage titer relied on observing and counting the obtained plaques^28^.

### Phage propagation

Phage lysate obtained from previously purified phage suspension was incubated with the host strain as described in the phage isolation, three successive times, using the phage lysate instead of the environmental samples^29^.

### Screening of the antibiofilm potential of the isolated phages

The previously isolated strong biofilm producers, *P. aeruginosa*, were allowed to form biofilm in 96-well plates under static conditions for 24 h at 37 °C. Then, bacterial suspensions were aspirated, followed by PBS washing twice to remove the planktonic cells. The isolated phage with an initial titer of 1.2 × 10^8^ PFU/ml was dispensed as 200 µL/well, followed by incubation for 24 h at 37 °C. The plate was PBS-washed and stained with CV, and the percentage reduction in biofilm formation was calculated compared to positive control wells (uninoculated with phage lysate)^30^. Each isolate was tested in an independent triplicate.

### Identification of the isolated phages showing lytic activities against *P. aeruginosa*

#### Morphology of the isolated bacteriophages

The morphological appearance of the isolated phages was preformed to classify the isolated phage using transmission electron microscope (TEM) through preparation of high titer purified phage lysate as described before and examining this lysate using TEM (Version JEOL_JEM_1400 Electron Microscope Nieuw-Vennep, Tokyo, Japan) located at Cairo University Research Park, Faculty of Agriculture, Cairo, Egypt. ImageJ software was used to measure the head and tail lengths from TEM images of the phage lysates^31^.

#### Extraction of phage genetic material and genomic sequencing

The DNA was extracted, quantified, and then sequenced, followed by data analysis as follows. DNA was extracted from the phage lysate using the DNA Isolation Kit (Norgen, Cat no. 46800) according to the manufacturer’s instructions. DNA quantification was carried out using Qubit 4 (Thermo Fischer Scientific), where 200 ng were applied for library preparation using Rapid sequencing DNA V14 - barcoding kit (SQK-RBK114.24; Oxford Nanopore Technologies, Oxford, UK) as recommended by the manufacturer’s protocol. Genome sequencing was carried out using MinION™ and R10.4.1 flow cells (FLO‐MIN114; Oxford Nanopore Technologies). Finally, the MinKNOW software version. 24.11.10 (Oxford Nanopore Technologies) was applied for data acquisition.

### Characterization of the isolated phages showing lytic activities against *P. aeruginosa*

#### Host range

The host range was performed in order to evaluate the spectrum of the activity of the isolated phage against different bacterial isolates using a spot test. A previously prepared TSA base layer overlaid with 0.7% TSA layer was inoculated with the tested bacterial isolates. Then 15 µL of each phage lysate was spotted on the top of each plate, and the plates were incubated overnight in an upright position. Following incubation, the formed inhibition zones were observed visually^32^.

#### Formulation of phage-loaded carboxy methyl cellulose (CMC)-hydrogel

Regarding the hydrogel formulation, 5% w/v CMC powder was dissolved gradually in distilled water to reach a final volume of 100 mL. The pH was adjusted to 7.7, and the spreadability coefficient was adjusted to 25. The stirring process continued till the formation of a transparent gel. The prepared hydrogel was autoclaved at 121 °C for 15 min and stored for 24 h at room temperature to stabilize. Finally, 1 mL (1.2 × 10^8^ PFU/mL) of the purified phage was mixed with 9 mL of the hydrogel base^12^.

### In vitro antibacterial potential of phage-loaded CMC-hydrogel

#### Inhibition zone assay

The ability of the phage to maintain its lytic activity when it is incorporated into the prepared hydrogel polymer was evaluated using a spot test, where the appearance of an inhibition zone predicts the activity of the phage hydrogel. A phage-free hydrogel was applied as a negative control, whereas phage lysate served as a positive control. In brief, 15 µL of different samples were spotted on the bacterial lawn (0.5 McFarland) cultured on TSA plates, incubated at 37 °C for 24 h, and tested for clear zone formation on the bacterial lawn^19^. Also, to evaluate its stability, the phage-loaded hydrogel was refrigerated at 4 °C for three months and examined once per week for three successive months to test for the maintenance of its lytic activity using a spot test.

### In vivo antibacterial potential of the phage-loaded hydrogel

The rat thermal injury model was used to evaluate the in vivo activity of the phage-loaded hydrogel. A total of 24 Swiss albino rats (weighing approximately 110–120 g) were used in the animal model. All animals were housed in open cages and fed an antibiotic-free diet consisting mainly of 20% protein, 6.5% ash, 5% fiber, and 3.5% fat, with free access to water. They were kept on an alternating 12 h light-dark cycle and a constant temperature of 25 °C adjusted by an air conditioner. Animals were maintained in accordance with the Care and Use of Laboratory Animals recommendations and ARRIVE guidelines (https://arriveguidelines.org). The study was approved by the Faculty of Pharmacy, Ain Shams University, Ethics Committee, ACUC-FP-ASU REC# 339.

### Thermal injury model

Third-degree burn was induced according to El-Kased et al.^33^ with a minor modification. The back of each animal, after shaving and using a hot metal bar (1 × 1 cm and 1 mm thickness), was heated for 30 s using an open flame and placed on the shaved back of the rat skin for 20 s. Animals were classified according to Mabrouk et al.^12^ into four groups: Group I: normal control, intact, non-infected, untreated. Group II: negative control, burned, infected, untreated. Group III: positive control, Burned, infected, treated with silver burn cream. Group IV: Test, burned, infected, treated with tested phage-loaded hydrogel.

### Treatment

Two hours post-infection, the treatment with the tested hydrogel and standard drug was initiated on the burned infected area by applying approximately 1 g of the tested hydrogel and the cream topically. The dose regimen was adjusted to be twice daily for 14 days for both the tested hydrogel and the standard drug. The evaluated parameters were the survival rate of animals (monitored for 14 days post-infection), and the viable bacterial load was counted at the infected wound area using a sterile swab. For counting the viable bacterial cells, sterile swabs were moistened using sterile saline and swabbed along a limited infected area of the wound, and then immersed in 1 ml of sterile saline, followed by vortexing mildly to release bacteria^34^. Finally, the animals were anaesthetized intraperitoneally with a cocktail of ketamine (60 mg kg-1) and xylazine (12 mg kg-1) before being euthanized by cervical dislocation^35^. Then, the dorsal skin section from the wound area of all rates was immediately removed for histopathological examination. The total sample size was 24.

### Wound area closure

The wound area was measured in mm using a ruler on days 0, 6, and 14. These measurements were documented to follow up on the wound area closure and the progress of wound healing among the experimental groups^33^. Then the wound contraction percentage was calculated as follows: Wound Contraction (%) = (A_0_ - A_t_ /A_0_) *100.

where A_0_ = The initial wound area measured on day 0 (10 mm^2^). At = The wound area measured on a specific day on days 7 and 14^36^.

### Histopathological examination

The collected skin tissue sections were fixed in formalin for 24 h before examination. The fixed tissue samples were processed for the hematoxylin and eosin (H&E) staining for tissue histological features, inflammatory infiltrations, and healing progression. Masson Trichrome staining was carried out to evaluate collagen deposition. This process was performed under the supervision of the Pathology department at the Faculty of Medicine, Mansoura University.

### Statistical analysis

The bacterial load recovered from the infected wounds (log10 CFU/ml) was determined among four groups. Data was analyzed by one-way analysis of variance (ANOVA) using GraphPad Instat-3 software (GraphPad Software Inc., USA). Results were considered significant at p-value < 0.05 and presented as means ± standard deviation. Each experiment was performed in triplicate.

## Supplementary Information

Below is the link to the electronic supplementary material.

Supplementary Material 1

## Data Availability

All data generated or analyzed during this study are included in this published article in the main manuscript and the supplementary file.

## References

[1] Krell, T. & Matilla, M. A. Pseudomonas aeruginosa. *Trends Microbiol.* **32**, 216–218 (2024). 38065787 10.1016/j.tim.2023.11.005

[2] Zsila, F., Ricci, M., Szigyártó, I. C. & Singh, P. Beke-Somfai, T. Quorum sensing pseudomonas quinolone signal forms chiral supramolecular assemblies with the host defense peptide LL-37. *Front. Mol. Biosci.* **8**, 742023 (2021). 34708076 10.3389/fmolb.2021.742023 PMC8542694

[3] McVay, C. S., Velásquez, M. & Fralick, J. A. Phage therapy of Pseudomonas aeruginosa infection in a mouse burn wound model. *Antimicrob. Agents Chemother.* **51**, 1934–1938 (2007). 17387151 10.1128/AAC.01028-06 PMC1891379

[4] Ma, J. et al. vB_PaeP_PZH3, a novel bacteriophage for the treatment of MDR Pseudomonas aeruginosa in a mouse wound infection model. *J. Glob Antimicrob. Resist.* **44**, 197–206 (2025). 40588032 10.1016/j.jgar.2025.06.019

[5] Reynolds, D. & Kollef, M. The Epidemiology and Pathogenesis and Treatment of Pseudomonas aeruginosa Infections: An Update. *Drugs* **81**, 2117–2131 (2021). 34743315 10.1007/s40265-021-01635-6 PMC8572145

[6] Moser, C. et al. Immune responses to pseudomonas aeruginosa biofilm infections. *Front. Immunol.* **12**, 625597 (2021). 33692800 10.3389/fimmu.2021.625597 PMC7937708

[7] Thi, M. T. T., Wibowo, D. & Rehm, B. H. A. Pseudomonas aeruginosa biofilms. *Int. J. Mol. Sci.* **21**, 8671 (2020). 33212950 10.3390/ijms21228671 PMC7698413

[8] Fernandes, S. & São-José, C. Enzymes and mechanisms employed by tailed bacteriophages to breach the bacterial cell barriers. *Viruses* **10**, 396 (2018). 30060520 10.3390/v10080396 PMC6116005

[9] Zyman, A., Górski, A. & Międzybrodzki, R. Phage therapy of wound-associated infections. *Folia Microbiol. (Praha)*. **67**, 193–201 (2022). 35028881 10.1007/s12223-021-00946-1 PMC8933295

[10] Sawa, T., Moriyama, K. & Kinoshita, M. Current status of bacteriophage therapy for severe bacterial infections. *J. Intensive Care*. **12**, 44 (2024). 39482746 10.1186/s40560-024-00759-7 PMC11529433

[11] Zalewska-Piątek, B. & Nagórka, M. Phages as potential life-saving therapeutic option in the treatment of multidrug-resistant urinary tract infections. *Acta Biochim. Pol.* **72**, 14264 (2025). 40007695 10.3389/abp.2025.14264 PMC11850123

[12] Mabrouk, S., Abdellatif, G. R., Abu Zaid, A. S., Aziz, R. K. & Aboshanab, K. M. In vitro and pre-clinical evaluation of locally isolated phages, vB_Pae_SMP1 and vB_Pae_SMP5, formulated as hydrogels against carbapenem-resistant pseudomonas aeruginosa. *Viruses* **14**, 2760 (2022). 36560763 10.3390/v14122760 PMC9780878

[13] Gutiérrez, D., Ruas-Madiedo, P., Martínez, B., Rodríguez, A. & García, P. Effective removal of staphylococcal biofilms by the endolysin LysH5. *PLoS One*. **9**, e107307 (2014). 25203125 10.1371/journal.pone.0107307 PMC4159335

[14] Abdelrahman, F. et al. Phage-encoded endolysins. *Antibiot. (Basel)*. **10**, 124 (2021). 10.3390/antibiotics10020124 PMC7912344 33525684

[15] Shi, Z. et al. Characterization of the novel broad-spectrum lytic phage Phage_Pae01 and its antibiofilm efficacy against Pseudomonas aeruginosa. *Front. Microbiol.* **15**, 1386830 (2024). 39091310 10.3389/fmicb.2024.1386830 PMC11292732

[16] Topka-Bielecka, G. et al. Bacteriophage-derived depolymerases against bacterial biofilm. *Antibiot. (Basel)*. **10**, 175 (2021). 10.3390/antibiotics10020175 PMC7916357 33578658

[17] Zhang, S. et al. Characterization of two novel bacteriophages (PSV6 and PSV3) as biocontrol agents against Pseudomonas syringae. *Front. Microbiol.* **16**, 1633072 (2025). 41311495 10.3389/fmicb.2025.1633072 PMC12651447

[18] Mohamed, A. A., El-Zayat, E. M. & El-Shibiny, A. Efficacy of phage vB_Ps_ZCPS13 in controlling Pan-drug-resistant Pseudomonas aeruginosa from urinary tract infections (UTIs) and eradicating biofilms from urinary catheters. *Virol. J.* **22**, 236 (2025). 40652263 10.1186/s12985-025-02848-x PMC12255097

[19] Sherif, M. M., Abdelaziz, N. A., Alshahrani, M. Y., Saleh, S. E. & Aboshanab, K. M. In vitro, genomic characterization and pre-clinical evaluation of a new thermostable lytic Obolenskvirus phage formulated as a hydrogel against carbapenem-resistant Acinetobacter baumannii. *Sci. Rep.* **15**, 17149 (2025). 40382448 10.1038/s41598-025-99788-x PMC12085604

[20] Shafigh Kheljan, F. et al. Design of phage-cocktail-containing hydrogel for the treatment of Pseudomonas aeruginosa-infected wounds. *Viruses* **15**, 803 (2023). 36992511 10.3390/v15030803 PMC10051971

[21] Wang, J. et al. Topically applied bacteriophage to control multi-drug resistant Pseudomonas aeruginosa-infected wounds in a New Zealand rabbit model. *Front. Microbiol.* **13**, 1031101 (2022). 36329839 10.3389/fmicb.2022.1031101 PMC9624279

[22] Ross, A., Ward, S. & Hyman, P. More is better: Selecting for broad host range bacteriophages. *Front. Microbiol.* **7**, 1352 (2016). 27660623 10.3389/fmicb.2016.01352 PMC5014875

[23] Chung, K. M., Liau, X. L. & Tang, S. S. Bacteriophages and their host range in multidrug-resistant bacterial disease treatment. *Pharmaceuticals (Basel)*. **16**, 1467 (2023). 37895938 10.3390/ph16101467 PMC10610060

[24] Chimi, L. Y., Noubom, M., Bisso, B. N., Njateng, S. & Dzoyem, J. P. G. S. Biofilm formation, pyocyanin production, and antibiotic resistance profile of Pseudomonas aeruginosa isolates from wounds. *Int J Microbiol* 1207536 (2024). (2024). 10.1155/2024/1207536 PMC10898945 38414729

[25] Humphries, R., Bobenchik, A. M., Hindler, J. A. & Schuetz, A. N. Overview of changes to the clinical and laboratory standards institute performance standards for antimicrobial susceptibility testing, M100, 31st edition. *J. Clin. Microbiol.* **59**, e0021321 (2021). 34550809 10.1128/JCM.00213-21 PMC8601225

[26] Stepanović, S. et al. Quantification of biofilm in microtiter plates: overview of testing conditions and practical recommendations for assessment of biofilm production by staphylococci. *APMIS* **115**, 891–899 (2007). 17696944 10.1111/j.1600-0463.2007.apm_630.x

[27] Sherif, M. M., Elkhatib, W. F., Khalaf, W. S., Elleboudy, N. S. & Abdelaziz, N. A. Multidrug resistant acinetobacter baumannii biofilms: Evaluation of phenotypic-genotypic association and susceptibility to cinnamic and gallic acids. *Front. Microbiol.* **12**, 716627 (2021). 34650528 10.3389/fmicb.2021.716627 PMC8508616

[28] Youssef, R. A., Sakr, M. M., Shebl, R. I., Saad, B. T. & Aboshanab, K. M. Genomic characterization, in vitro, and preclinical evaluation of two microencapsulated lytic phages VB_ST_E15 and VB_ST_SPNIS2 against clinical multidrug-resistant Salmonella serovars. *Ann. Clin. Microbiol. Antimicrob.* **23**, 17 (2024). 38360595 10.1186/s12941-024-00678-3 PMC10870556

[29] Abd-Allah, I. M. et al. An Anti-MRSA phage from raw fish rinse: Stability evaluation and production optimization. *Front. Cell. Infect. Microbiol.* **12**, 904531 (2022). 35656033 10.3389/fcimb.2022.904531 PMC9152141

[30] Abo Kamer, A. M., Abdelaziz, A. A., Nosair, A. M. & Al-Madboly, L. A. Characterization of newly isolated bacteriophage to control multi-drug resistant Pseudomonas aeruginosa colonizing incision wounds in a rat model: in vitro and in vivo approach. *Life Sci.* **310**, 121085 (2022). 36265569 10.1016/j.lfs.2022.121085

[31] Glonti, T. & Pirnay, J. P. In vitro techniques and measurements of phage characteristics that are important for phage therapy success. *Viruses* **14**, 1490 (2022). 35891470 10.3390/v14071490 PMC9323186

[32] Camens, S. et al. Preclinical development of a bacteriophage cocktail for treating multidrug resistant Pseudomonas aeruginosa infections. *Microorganisms* **9**, 2001 (2021). 34576896 10.3390/microorganisms9092001 PMC8464757

[33] El-Kased, R. F., Amer, R. I., Attia, D. & Elmazar, M. M. Honey-based hydrogel: In vitro and comparative In vivo evaluation for burn wound healing. *Sci. Rep.* **7**, 9692 (2017). 28851905 10.1038/s41598-017-08771-8 PMC5575255

[34] Lagler, H. et al. Comparison of non-invasive Staphylococcus aureus sampling methods on lesional skin in patients with atopic dermatitis. *Eur. J. Clin. Microbiol. Infect. Dis.* **41**, 245–252 (2022). 34734346 10.1007/s10096-021-04365-5 PMC8770445

[35] Alrouji, M. et al. A simple in-vivo method for evaluation of antibiofilm and wound healing activity using excision wound model in diabetic Swiss Albino mice. *Microorganisms* **11**, 692 (2023). 36985266 10.3390/microorganisms11030692 PMC10051147

[36] Andritoiu, C. V. et al. Evaluation of the wound healing potential of some natural polymers on three experimental models. *Pharmaceuticals (Basel)*. **14**, 465 (2021). 34069274 10.3390/ph14050465 PMC8156046

