# Supplementary material for: Characterization and evaluation of the efficacy of phage E21 therapy in a wound animal model of biofilm-associated Pseudomonas aeruginosa infection
Source: Sci Rep. 2026 May 19;16:15490. doi: 10.1038/s41598-026-52857-1 (PMC13186941; doi:10.1038/s41598-026-52857-1)
Supplement: Supplementary file 1 — Supplementary Material 1 [file 41598_2026_52857_MOESM1_ESM.doc]

Supplementary Table S1.

Table 1. Clinical isolates, source of infection, sample type, and biofilm classification,

| **Isolate ID** | **Source of infection** | **Sample type** | **Hospital/Unit** | **Antibiotic resistance profile** | **Mean OD ± SD (630 nm)** | **Biofilm category** |
| --- | --- | --- | --- | --- | --- | --- |
| 1PS | Burn wound |  | Burn unit | MDR | 0.837 ± 0.026 | Strong |
| 2PS | Wound infection | Swab | Surgery ward | MDR | 0.888 ± 0.043 | Strong |
| 3PS | Wound infection | Swab | Surgery ward | MDR | 0.950 ± 0.040 | Strong |
| 4PS | Wound infection | Swab | Surgery ward | MDR | 0.898 ± 0.034 | Strong |
| 5PS | Wound infection | Swab | Surgery ward | MDR | 0.300 ± 0.007 | Moderate |
| 6PS | Wound infection | Swab | Surgery ward | MDR | 0.991 ± 0.024 | Strong |
| 7PS | Wound infection | Swab | Surgery ward | MDR | 0.968 ± 0.021 | Strong |
| 8PS | Pus Sample | Swab | Outpatient clinic | MDR | 0.875 ± 0.040 | Strong |
| 9PS | Pus Sample | needle | Outpatient clinic | MDR | 0.180 ± 0.007 | Weak |
| 10PS | Wound infection | Swab | Surgery ward | MDR | 0.323 ± 0.013 | Moderate |
| 11PS | Burn wound | Swab | Burn unit | MDR | 0.889 ± 0.018 | Strong |
| 12PS | Burn wound | Swab | Burn unit | MDR | 0.953 ± 0.047 | Strong |
| 13PS | Wound infection | Swab | Surgery ward | MDR | 0.160 ± 0.007 | Weak |
| 14PS | Wound infection | Swab | Surgery ward | MDR | 0.358 ± 0.009 | Moderate |
| 15PS | Burn wound | Swab | Burn unit | MDR | 0.938 ± 0.024 | Strong |
| 16PS | Burn wound | Swab | Burn unit | MDR | 0.859 ± 0.022 | Strong |
| 17PS | Burn wound | Swab | Burn unit | MDR | 0.180 ± 0.005 | Weak |
| 18PS | Wound infection | Swab | Surgery ward | MDR | 0.790 ± 0.028 | Strong |
| 19PS | Pus Sample | Swab | Outpateint clinic | MDR | 0.250 ± 0.008 | Moderate |
| 20PS | Pus Sample | Swab | Outpatient clinic | MDR | 1.090 ± 0.031 | Strong |
| 21PS | Wound infection | Swab | Surgery ward | MDR | 0.360 ± 0.014 | Strong |
| 22PS | Wound infection | Swab | Surgery ward | MDR | 0.400 ± 0.010 | Strong |
| 23PS | Wound infection | Swab | Surgery ward | MDR | 0.941 ± 0.027 | Strong |
| 24PS | Burn wound | Swab | Burn unit | MDR | 0.916 ± 0.028 | Strong |
| 25PS | Burn wound | Swab | Burn unit | MDR | 0.700 ± 0.024 | Strong |
| 26PS | Burn wound | Swab | Burn unit | MDR | 0.841 ± 0.037 | Strong |
| 27PS | Pus Sample | Swab | Outpatient clinic | MDR | 0.991 ± 0.026 | Strong |
| 28PS | Burn wound | Swab | Burn unit | MDR | 0.873 ± 0.031 | Strong |
| 29PS | Burn wound | Swab | Burn unit | MDR | 0.920 ± 0.035 | Strong |
| 30PS | Burn wound | Swab | Burn unit | MDR | 0.309 ± 0.007 | Moderate |
| 31PS | Burn wound | Swab | Burn unit | MDR | 0.500 ± 0.019 | Strong |
| 32PS | Wound infection | Swab | Surgery ward | MDR | 0.200 ± 0.005 | Moderate |
| 33PS | Wound infection | Swab | Surgery ward | MDR | 0.845 ± 0.019 | Strong |
| 34PS | Wound infection | Swab | Surgery ward | MDR | 0.934 ± 0.045 | Strong |
| 35PS | Wound infection | Swab | Surgery ward | MDR | 0.957 ± 0.047 | Strong |
| 36PS | Wound infection | Swab | Surgery ward | MDR | 0.891 ± 0.039 | Strong |
| 37PS | Wound infection | Swab | Surgery ward | MDR | 0.960 ± 0.028 | Strong |
| 38PS | Wound infection | Swab | Surgery ward | MDR | 0.985 ± 0.023 | Strong |
| 39PS | Wound infection | Swab | Surgery ward | MDR | 0.905 ± 0.037 | Strong |
| 40PS | Wound infection | Swab | Surgery ward | MDR | 0.270 ± 0.009 | Moderate |

Supplementary Table S2

Table S2: Genomic annotation analysis and the resulting open reading frames (ORFs) of Pseudomonas phage E21.

| **Start location** | **Stop location** | **Strand** | **Protein product** | **Protein function** |
| --- | --- | --- | --- | --- |
| 1 | 1128 | - | Phage portal protein, lambda family | infection; |
| 1128 | 1403 | - | GpW | assembly; |
| 1416 | 3482 | - | Phage terminase large subunit (GpA) | packaging;assembly; |
| 3475 | 4059 | - | Protein of unknown function (DUF1441) | unsorted; |
| 4062 | 5585 | - | helicase activity | replication; |
| 5545 | 5832 | - | hydrolase activity, acting on ester bonds | unsorted; |
| 5832 | 7865 | - | DNA polymerase family A | replication; |
| 7930 | 8535 | - | Protein of unknown function (DUF2815) | unsorted; |
| 8567 | 9895 | - | Protein of unknown function (DUF2800) | unsorted; |
| 9956 | 10438 | - | unknown | unsorted; |
| 10791 | 11075 | + | unknown | unsorted; |
| 11075 | 13750 | + | hydrolase activity, acting on acid anhydrides | unsorted; |
| 15160 | 15399 | - | unknown | unsorted; |
| 15396 | 15974 | - | unknown | unsorted; |
| 16198 | 16887 | - | unknown | unsorted; |
| 16940 | 17731 | - | unknown | unsorted; |
| 17893 | 18114 | - | unknown | unsorted; |
| 18098 | 18748 | - | Mom-like DNA modification protein | unsorted; |
| 18752 | 19492 | - | unknown | unsorted; |
| 20400 | 20969 | - | unknown | unsorted; |
| 21076 | 21477 | - | unknown | unsorted; |
| 21458 | 21649 | - | tail protein | infection; |
| 21636 | 21878 | - | unknown | unsorted; |
| 21890 | 22216 | - | unknown | unsorted; |
| 22686 | 23117 | + | hypothetical protein | hypothetical; |
| 23199 | 23510 | + | unknown | unsorted; |
| 23516 | 23836 | + | unknown | unsorted; |
| 23830 | 24177 | + | unknown | unsorted; |
| 24164 | 25252 | + | Putative exonuclease, RdgC | packaging; |
| 25522 | 26340 | + | unknown | unsorted; |
| 26337 | 27104 | + | unknown | unsorted; |
| 27101 | 27547 | + | unknown | unsorted; |
| 27540 | 28172 | + | unknown | unsorted; |
| 28172 | 28909 | + | DNA N-6-adenine-methyltransferase (Dam) | immune; |
| 29211 | 29744 | + | unknown | unsorted; |
| 29737 | 29988 | + | unknown | unsorted; |
| 29990 | 30859 | + | Unknown | unsorted; |
| 30856 | 31320 | + | nucleoside 2-deoxyribosyltransferase | immune; |
| 31570 | 31824 | - | Rz-like spanin | lysis; |
| 31824 | 32528 | - | Endolysin | lysis; |
| 32539 | 32853 | - | Rz-like spanin | lysis; |
| 32864 | 34849 | - | hypothetical protein | hypothetical; |
| 34864 | 35148 | - | Unknown | unsorted; |
| 35743 | 35931 | - | Unknown | unsorted; |
| 36253 | 36519 | - | Unknown | unsorted; |
| 36529 | 37545 | - | hypothetical protein | hypothetical; |
| 37558 | 38541 | - | hypothetical protein | hypothetical; |
| 38552 | 39472 | - | hypothetical protein | hypothetical; |
| 39472 | 40215 | - | Protein of unknown function (DUF2793) | unsorted; |
| 40215 | 44036 | - | Putative phage tail protein | infection; |
| 44029 | 44238 | - | tail assembly chaperone | assembly;infection; |
| 44238 | 44474 | - | tail assembly chaperone | assembly;infection; |
| 44487 | 45302 | - | Phage conserved hypothetical protein BR0599 | hypothetical; |
| 45324 | 47012 | - | tail assembly protein | assembly;infection; |
| 47018 | 49396 | - | peptidoglycan catabolic process | unsorted; |
| 49381 | 49761 | - | peptidoglycan catabolic process | unsorted; |
| 49733 | 51346 | - | peptidoglycan catabolic process | unsorted; |
| 51587 | 52039 | - | tail protein | infection; |
| 52178 | 53365 | - | major tail protein with Ig-like domain | infection; |
| 53369 | 53881 | - | tail terminator | infection; |
| 53871 | 54488 | - | virus tail | infection; |
| 54491 | 54844 | - | hypothetical protein | hypothetical; |
| 54844 | 55191 | - | hypothetical protein | hypothetical; |
| 55255 | 56322 | - | Phage major capsid protein E | assembly; |
| 56335 | 56751 | - | Bacteriophage lambda head decoration protein D | assembly; |
| 56765 | 58042 | - | Peptidase family S49 | lysis; |
| 58039 | 58554 | - | Phage portal protein, lambda family | infection; |

Supplementary Table S3

Table S4: Evaluation of the degree of wound healing in each experimental group along with time

|  | **Wound contraction (%)** | | |
| --- | --- | --- | --- |
| **Time point/Group** | Group II | Group III | Group IV |
| **Day 0** | 0% | 0% | 0% |
| **Day 7** | 0% | 30% | 50% |
| **Day 14** | 40% | 80% | 90% |

Group II: negative control, burned, infected, untreated; Group III: positive control, burned, infected, treated with silver burn cream. Group IV: Test, burned, infected, treated with phage -loaded hydrogel.

Supplementary Table S4

Table 4: Bacterial load detected in the infected wound area among different experimental groups**.**

| Time Point | Group I | Group II | Group III | Group IV |
| --- | --- | --- | --- | --- |
| Day 0 | 0±0.0 | 8.00±0.12 | 8.00±0.15 | 8.00±0.11 |
| Day 7 | 0±0.0 | 7.00±0.25 | 6.00±0.32 | 2.00±0.18 |
| Day 14 | 0±0.0 | 5.00±0.41 | 2.00±0.22 | 1.00±0.14 |

Group I (normal group): non-infected, untreated; Group II: negative control, burned, infected, untreated; Group III: positive control, burned, infected, treated with silver burn cream. Group IV: Test, burned, infected, treated with phage -loaded hydrogel. The experiment was carried out in triplicate, and results were expressed as (log_10_ CFU/unit ± SD) and indicated a statistically significant reduction (p = 0.0026) in the bacterial count in group IV (infected, treated with phage-loaded hydrogel) as compared to group III (infected, treated with silver sulfadiazine) on day 14

Supplementary Figure S1


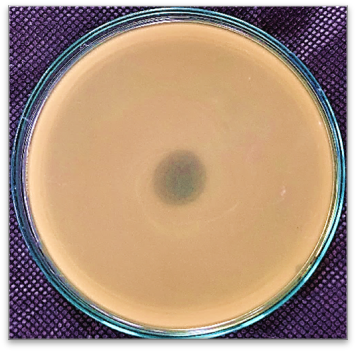


**Fig 1**. Uncropped picture for Figure 1a.

Supplementary Figure S2


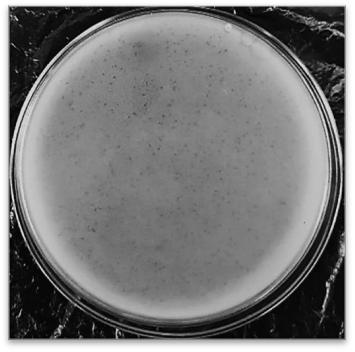


**Fig 2**. Uncropped picture for Figure 1b.

Supplementary Figure S3


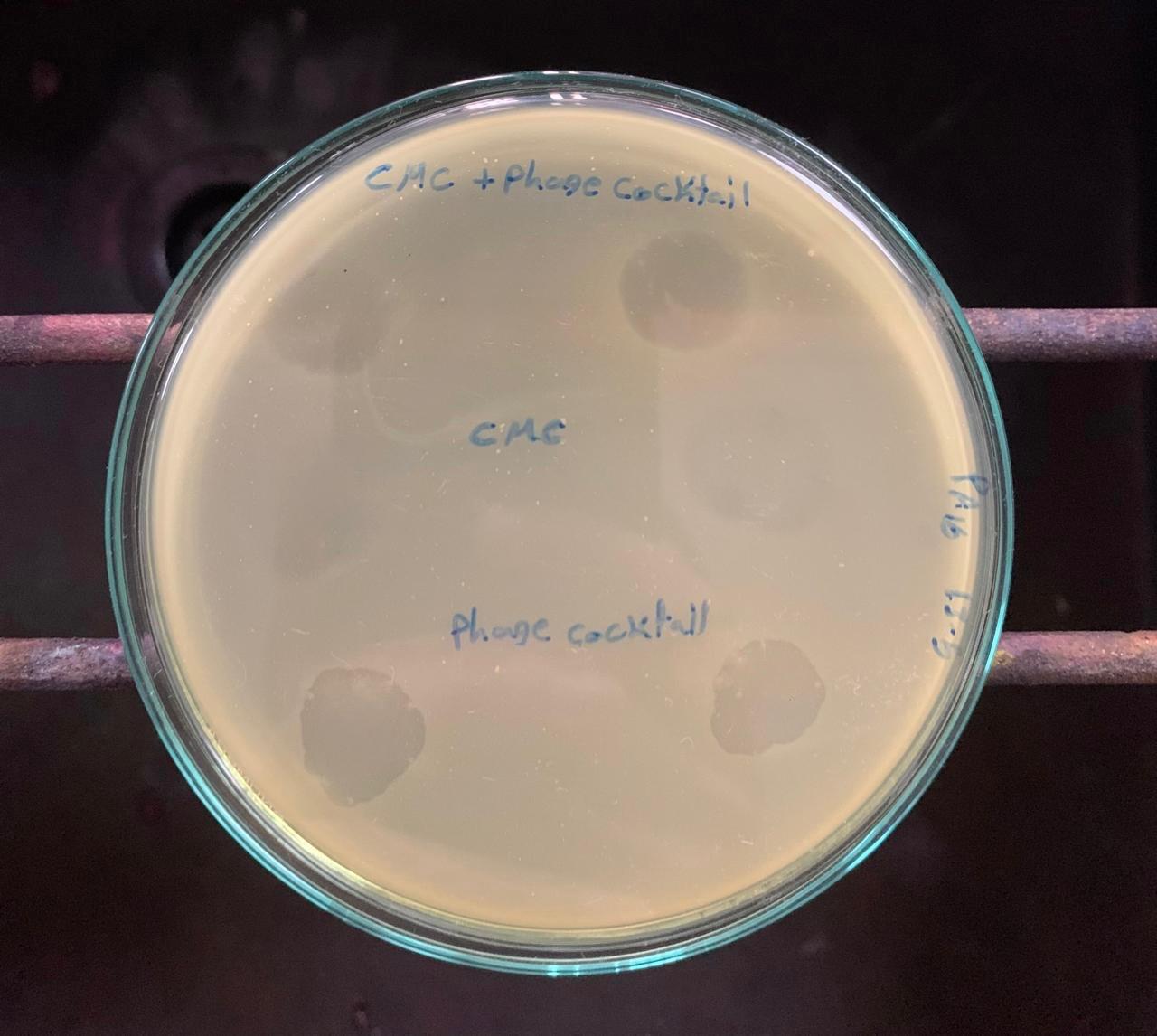


**a**

**b**

**c**

**Fig 3.** In vitro activity of the Pseudomonas phage E21-loaded hydrogel (a), negative control (b), and positive control (c) against a strong biofilm-forming *P. aeruginosa* clinical isolate.

Supplementary Figure S4


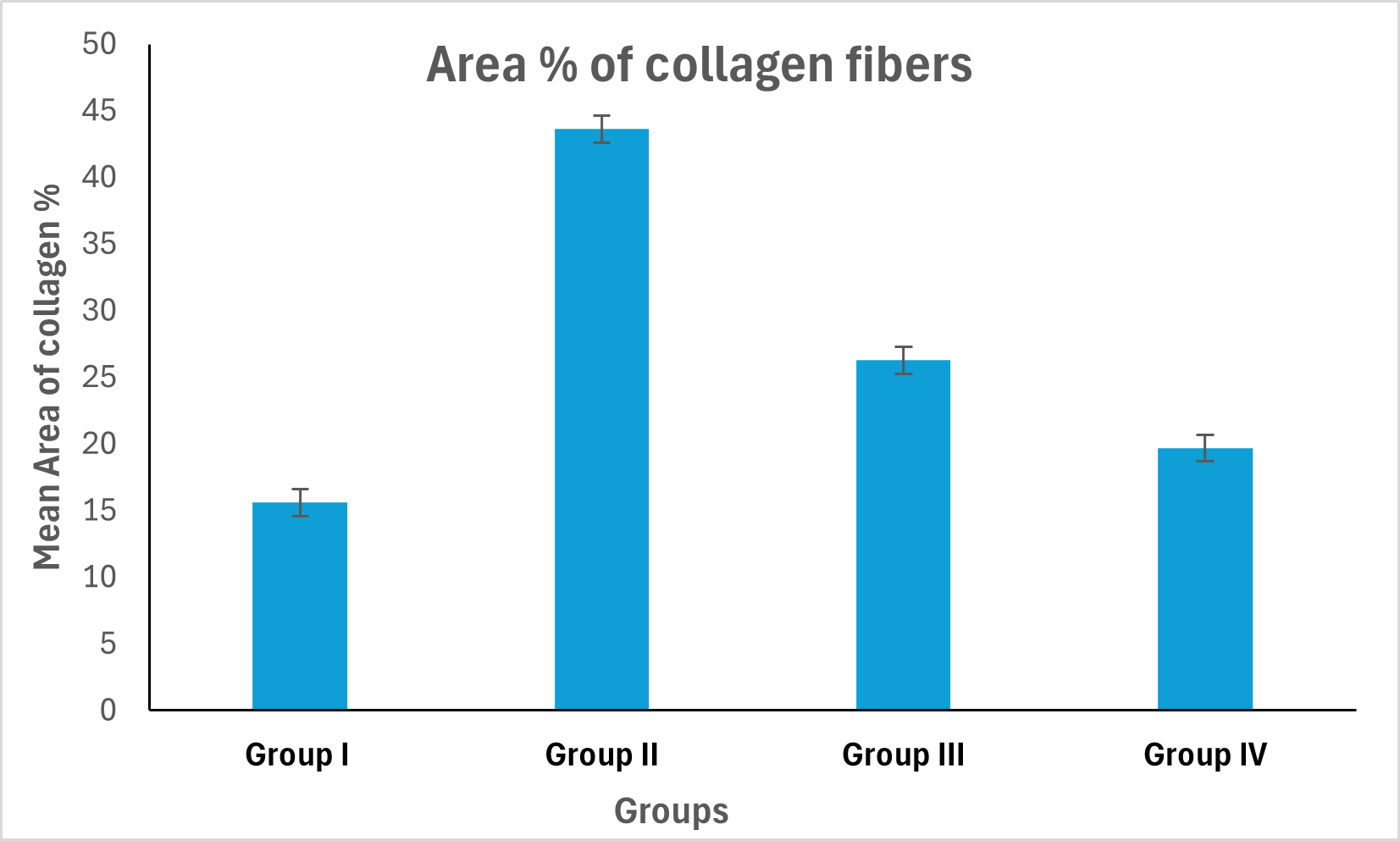


*

**Fig 4.** Mean area % of collagen fibers, data presented as mean ±SD. *: highly significant difference. One-way ANOVA was used to assess the statistical significance among the studied groups.
